# Feasibility of Text Messages for Enhancing Therapeutic Engagement Among Youth and Caregivers Initiating Outpatient Mental Health Treatment: Mixed Methods Study

**DOI:** 10.2196/35685

**Published:** 2022-08-02

**Authors:** Susan Jerrott, Sharon Clark, Jill Chorney, Aimee Coulombe, Lori Wozney

**Affiliations:** 1 Mental Health and Addictions Program Nova Scotia Health Dartmouth, NS Canada; 2 IWK Health Halifax, NS Canada; 3 Department of Psychiatry Dalhousie University Halifax, NS Canada

**Keywords:** text messaging, youth, mental health, waiting list, informatics, health behavior, self-care, mental health literacy, caregivers, transdiagnostic

## Abstract

**Background:**

Pathways to mental health services for youth are generally complex and often involve numerous contact points and lengthy delays. When starting treatment, there are a host of barriers that contribute to low rates of therapeutic engagement. Automated text messages offer a convenient, low-cost option for information sharing and skill building, and they can potentially activate positive behaviors in youth and caregivers prior to beginning formal therapy. To date, there is little evidence for the feasibility of initiating transdiagnostic text messages during the early stages of youth and caregiver contact with community outpatient mental health services.

**Objective:**

To develop and test the feasibility of implementing 2 novel text messaging campaigns aimed at youth clients and their caregivers during the early stages of engaging with outpatient mental health services.

**Methods:**

A multidisciplinary panel of experts developed two 12-message interventions with youth and caregivers prior to deployment. Each message included a link to an external interactive or multimedia resource to extend skill development. Enrollment of youth aged 13 to 18 years, their caregivers, or both occurred at 2 early treatment timepoints. At both time points, text messages were delivered automatically 2 times a week for 6 weeks. Analytics and survey data were collected in 2 phases, between January and March 2020 and between January and May 2021. Enrollment, willingness to persist in using the intervention, engagement, satisfaction, perceived value, and impact were measured. Descriptive statistics were used to summarize youth and caregiver outcomes.

**Results:**

A total of 41 caregivers and 36 youth consented to participate. Follow-up survey response rates were 54% (22/41) and 44%, (16/36) respectively. Over 1500 text messages were sent throughout the study. More than three-quarters (14/16, 88%) of youth reported that they learned something new and noticed a change in themselves due to receiving the texts; the same proportion (14/16, 88%) of youth said they would recommend the text messages to others. Youth ranked the first text message, related to coping with difficult emotions, as the most helpful of the series. Caregivers reported acting differently due to receiving the texts. Over two-thirds of caregivers were satisfied with the texts (16/22, 73%) and would recommend them to others (16/22, 73%). Caregivers perceived diverse levels of value in the text topics, with 9 of the 12 caregiver texts rated by at least one caregiver as the most helpful.

**Conclusions:**

Results are preliminary but show that brief, core skill–focused text messages for youth clients and caregivers in community outpatient mental health services are feasible. Both youth and caregivers reported promising knowledge and behavior change with exposure to only 12 messages over 6 weeks. A larger study with statistical power to detect changes in both perceived helpfulness and engagement is required to confirm the effectiveness of this type of transdiagnostic intervention.

## Introduction

Successfully addressing the mental health concerns of youth depends not only on timely help-seeking behavior, but also on a rapid and appropriate response by the mental health system [1,2]. Pathways to mental health services for youth are generally complex and often involve numerous contact points and delays [3]. Initial assessment does not guarantee referral to treatment, and many of those referred do not attend their first scheduled appointment [4]. It is increasingly clear that planned early interventions across initial system contact points, including preclinical contact, can help avoid downstream negative impacts (ie, worsening symptoms, lower treatment satisfaction, and no-shows) and build momentum for sustained engagement [5-7]. Even a single early destigmatizing experience may be sufficient to promote future help-seeking behavior in youth and even delay illness progression [8].

Transforming reluctance into investment in treatment requires establishing mutual understanding among clinicians, youth, and their caregivers about the purpose, goals, and actions needed by all involved for positive change in the client’s life [9]. With a growing body of literature showing that the constituent “practice elements” of evidence-based interventions can be relevant to a wide variety of child and youth mental health conditions (ie, they are not restricted to treatments for a specific type of problem or disorder) [10-13], there is an opportunity to automate universal low-intensity supports and make them part of routine service initiation pathways. Socializing youth and caregivers to help them learn transdiagnostic skills that target core-skill mechanisms (eg, strategies for emotional regulation, skills for dealing with positive and negative valence systems, problem solving, sleep regulation, self-identity, and communication skills) [14] could have significant positive individual and system level outcomes [15].

A recent scoping review of youth and family engagement research found that facilitating early access to complementary and digital mental health services is particularly desirable when wait times cannot be avoided, or when community conditions and geographic constraints prohibit routine engagement with outpatient services [16]. Short messaging service (SMS) or text messages offer a convenient, low-cost communication channel with which to establish connections with youth and their caregivers and activate transdiagnostic mental wellness strategies and skills. A scoping review by our team [17] showed that text messaging interventions are now used in many areas of child and youth mental health, including mild to moderate anxiety [18], bipolar disorder [19], appointment reminders [20], medication adherence [21], and mood disorders [22].

The literature shows high rates of acceptability among youth [23,24], with only a small percentage of users unsubscribing from therapeutic text messages [25] when given the opportunity. Thematic analysis of interviews with youth who presented to the emergency department for mental health treatment indicated that cognitive behavioral therapy–based messages resonated with them, as did emotional regulation messages [26]. In some instances, text message interventions have led to significant behavior change [27] and a decrease in symptoms [28]. Exploring the feasibility of using text messages to deliver transdiagnostic supportive messages to both caregivers and youth during early interactions with outpatient services is a novel contribution to this evolving field.

The intention of this study was to develop and assess a text messaging campaign to engage youth clients and their caregivers while initiating outpatient service pathways. Through sharing tips, self-care ideas, and skill-building strategies and promoting new behaviors, the text messages were viewed as an opportunity to empower youth and caregivers to activate self-management skills prior to initiation of formal therapy.

The specific questions guiding the study were: (1) How many youth clients and caregivers want to receive text messages to support their general wellness when initially offered? (2) Once signed up to receive the text messages, do youth or caregiver clients choose to stop receiving text messages? (3) Which topics or types of resources being communicated through text messages are youth and caregivers most interested in? (4) In what ways do youth and caregivers feel that the text messages are or are not helpful in supporting behavioral change and in preparing them for their next appointment?

## Methods

### Study Design

A multimethod, descriptive research design was used for formative feasibility testing. Web analytics and survey data were collected in 2 phases between January 2020 and May 2021.

### Text Message Development

The text message intervention was informed by an earlier cross-sector knowledge mobilization project involving several of the authors. That project sought to address local gaps in youth mental health supports using technologies such as text messaging [29]. In particular, the intervention aimed to focus on patient activation, which was defined as creating a common language for patients to share their concerns with health care providers; to learn about their condition to enable informed future decisions about treatment; to set expectations that they would take an active role in their own care; and to learn basic coping skills to manage symptoms [30]. Development and deployment of the text message intervention for youth and caregivers occurred in 5 steps, described in the following sections.

#### Step 1

Prioritization of content for the text messages was identified through the results of the team’s scoping review [17] and a team activity completed by community mental health and addictions team clinicians at IWK Health. Clinicians were asked to come up with a list of the most important core skills in working with local clients and families. To the degree that a given skill or activity (eg, relaxation techniques, facing fears, and sleep hygiene) targeted multiple disorders (eg, anxiety, depression, and conduct problems) the text messages were viewed as potentially transdiagnostic.

#### Step 2

A panel was created with 5 members who had a broad range of expertise, including clinical psychology, psychotherapeutic competencies, behavior change, digital health, and implementation science. The panel achieved early consensus on several key aspects of the text message interventions to be created: (1) include a web link to an outside resource for each text message topic, (2) minimize the burden on users by presenting a brief and engaging topical prompt for each text message, and (3) establish a time-limited messaging period with no more than twice-weekly messages being sent.

#### Step 3

Taking the list of important core skills identified in step 1, the panel engaged in a collaborative process to draft prototype versions of the text message intervention for youth and caregivers. The panel aimed to create messages appropriate for a broad spectrum of individual youth and caregiver experiences and needs. Each text message contained a short topical statement and a link to a further resource to deepen understanding, build skills, or reinforce concepts through interactive multimedia. The linked resources were all publicly available online and were vetted by the panel for clinical and developmental appropriateness and to ensure a variety of modalities (ie, not all videos or not all PDFs). Many of the links were resources familiar to clinicians on the team and already in use as informational resources that youth and caregivers might be directed to as part of treatment sessions.

#### Step 4

Drafts of the text messages and links were checked for face validity with 6 youth and 5 caregivers. The testers suggested rewording, deleting some proposed content areas, noted links that they did not like, and suggested resources and links. Each tester also ranked a series of text message topics to determine which 12 topics were the most salient to them. Feedback was incorporated, and the final list of messages was completed (Table 1). Examples of the text messages included: (1) (for teens) “Sometimes it feels good to let your feelings out. But don’t let yourself get overwhelmed by the ones you don’t want, like fear and rejection. Learning to control feelings can help a lot. Here’s some ideas on how to do that” and (2) (for caregivers) “Is your teen’s fear controlling your family? When worry starts to take control you need to coach your teen to face it: one step at a time.”

**Table 1:** Youth and caregiver message themes and description of resources.

Message number	Topic	Linked resources for youth	Linked resources for caregivers
1	Difficult emotions	Animated YouTube video about anxiety	Animated YouTube video about empathy
2	Facing fears	Handout on building a plan to face your fears	Activity on how to build a fear ladder
3	Calming anger	Handout with tips on reducing anger	Web article on emotional coaching
4	Sleep habits	Website with frequently asked questions on how to deal with sleep challenges	Web article with tips for talking to youth about sleep
5	Self-care strategies	Interactive website with searchable self-care activity library	Guidebook for caregivers including tips for caregiver self-care
6	Relaxation skills	Animated video on breathing exercises	Web article on how to dial back conflict at home
7	Problem-solving	Structured problem-solving worksheet	Structured problem-solving worksheet
8	Negative thinking	Visual comparing negative versus realistic thinking	Web article on responding to youth’s negative self-talk
9	Behavioral activation	Catalogue of 365 fun activity ideas	Catalogue of 365 fun activity ideas
10	Mindfulness	Animated, guided mindfulness exercise	Website with >40 downloadable podcasts
11	Values	Animated YouTube video on finding your own happiness	Animated YouTube video on finding your own happiness
12	Social media	Infographic on how to hold a “digital detox”	Blog post on being a digital mentor to your teen

#### Step 5

The text messages were set up for automated delivery using an SMS platform (SimplyCast) [31]. The platform provided a user-friendly interface to build text message campaigns, embed study surveys, and track user engagement. SimplyCast utilizes firewalls, cryptography, and access-control procedures to protect information from unauthorized access. SimplyCast is ISO 27001, 27017, and 27018 certified. All information within the SimplyCast platform is stored on servers located in Canada. Text messages were set up to be automatically sent out at 6:00 PM, 2 times per week (3 days apart), for 6 weeks. This time of delivery was chosen because youth and caregivers were more likely to be out of school or work at that time, thus increasing the probability that they could engage with the linked resource. Participants could text “STOP” at any time to opt out of receiving the text messages. After the participant received their 12th therapeutic text message, a final message provided an embedded direct link to a brief web-based follow-up survey.

### Setting

**Figure 1:**
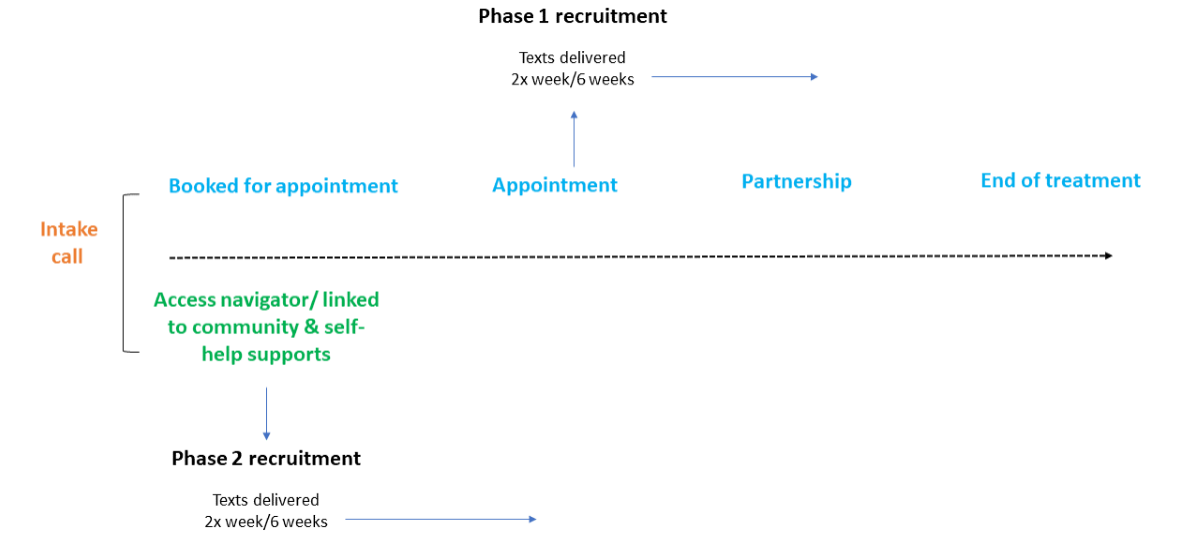
Process flow diagram for text message delivery within the treatment pathway.

The IWK community mental health and addictions teams use the choice and partnership approach (CAPA) to outpatient child and youth mental health and addictions treatment. CAPA is a service transformation model that includes active collaboration with clients and families, lean thinking concepts, and therapeutic engagement tasks [32]. After an initial intake call, the family is either booked for a choice appointment with a clinician or receives support from an access navigator to connect directly with community resources appropriate to meet their mental wellness needs (Figure 1). The choice appointment is a time for collaboratively developing a plan with the youth, family, and clinician for next steps that could be helpful to meet their mental health goals, regardless of whether returning for clinical treatment sessions is part of that plan. Based on the identified goals, options for next steps may include self-help information or helping the youth and family to access relevant supports within or external to the outpatient community mental health services. A fundamental stance of the CAPA way of working is to value the expertise of the youth and family and engage them in actively participating in goal setting and decision-making. Administrative data from 2019 show that approximately 1987 youth were referred for a choice appointment after their intake call that year. Youth who have mental health treatment needs are offered an opportunity to book for treatment (partnership) after the choice appointment.

The 2-phase recruitment plan for this study was partially driven by real-time data monitoring. Initially, in 2019, the wait time between the choice appointment and the partnership ranged from 15 to 76 days (Figure 1); this presented an important window, where contact via text message could help bridge the lapse in contact with outpatient clinicians. In the second phase, during the early months of 2021 (January to March) we anticipated a potential influx of referrals due to the pandemic; data showed that the wait for choice appointments ranged from 36 to 39 days.

### Population

Census data related to the IWK catchment area show that the community from which the study participants were recruited could be characterized as urban, predominantly English speaking, and White; 17% of the population are in households considered to have low incomes [33]. Participants were youth, aged 13 to 18 years, and their caregivers, who had inquired about services from the IWK community mental health and addictions program. Participants did not have to sign up as a youth-caregiver dyad, to ensure access to anyone who was interested. In this way, youth could still participate even if a caregiver did not attend the appointment. Youth clients and caregivers were eligible if they spoke English, had a cell phone, could read text messages, and were willing to receive up to 12 messages over a 6-week period.

### Recruitment and Informed Consent

Recruitment occurred in 2 phases. In phase 1 (January to March 2020), clinicians described the study to families while they were facilitating a choice appointment and helped those interested to sign up while in the office, before going home. If youth came to appointments without a caregiver, only the youth was offered the service. Clients and caregivers signed up by texting a short code (ie, IWKTEEN or IWKCAREGIVER) to a specific number. Following the short code, an automated welcome text message with an embedded web-based consent form was sent. In phase 2 (January 2021 to May 2021), access navigators on the intake team described the study to the youth or caregivers at the end of their intake phone call and offered to assist those interested in signing up during the call. A welcome text message with an embedded web-based consent form was sent, as in phase 1. No remuneration was offered to those participating.

### Measures

By reviewing the existing literature on outcomes associated with text messaging interventions for youth mental health [17], discussing what outcomes were of interest to local decision-makers, and aligning the outcome measures to the purpose of a pilot study (ie, to identify potential effects and associations to explore in a larger study) we selected a range of exploratory engagement, impact, and satisfaction measures.

#### Uptake

In phase 1 only, clinicians in 3 outpatient clinics tracked the number of youth and caregivers who were offered and subsequently enrolled in the text messaging service. We calculated the percentage of those who accepted as a preliminary measure of uptake.

#### Willingness to Use the Service

In addition to the data tracked about participant recruitment and enrollment, we tracked instances when participants did not desire to continue using the service. Dropouts (ie, those who texted “STOP”) were recorded.

#### Engagement

To measure engagement with the text messages, we calculated the total overall number of times each link within a message was clicked or reclicked.

#### Satisfaction

A 10-item follow-up survey was used that included 2 satisfaction questions. A global measure was ascertained by asking users to rate their level of satisfaction with the text messages on a 5-point Likert scale: “very dissatisfied,” “slightly dissatisfied,” “neutral,” “slightly satisfied,” and “very satisfied” (item # 1). A second question asked about satisfaction with the text message frequency (item #2).

#### Value

Four survey questions assessed perceived value. A global measure was assessed by asking users to rate the text message intervention on a 5-point Likert scale of helpfulness: “not helpful at all,” “somewhat helpful,” “neutral,” “helpful,” and “very helpful” (item #3). In addition, participants were asked to identify which 1 of the 12 text messages was the most helpful (item #4) and which was the least helpful (item #5). One additional question asked respondents if they would recommend the text messages to someone they knew (item #7).

#### Impact

Four questions on the survey measured impact. One asked about changes in knowledge; in other words, if the user learned anything from receiving the text messages (item # 8). Another asked if the user found themselves acting differently or changing their behavior after receiving the text messages (item #9) or noticed any changes in themselves as a result of receiving the text messages (item #6). Lastly, during phase 1, we asked the users if the text messages helped ready them for their next step in the therapeutic pathway (ie, attending their partnership appointment) (item #10).

### Analysis

As the underlying parameters of the interventions were the same across recruitment phases (same messages, delivery schedule, and links), data from both phases of recruitment were pooled. Descriptive statistics appropriate for the level of measurement were conducted using Jeffreys’s Amazing Statistics Program (JASP) [34]. Frequency counts and percentages were used to summarize the results. Graphs were used to examine engagement over time.

### Ethics Approval

Institutional review board approval was obtained from the IWK Health Research Ethics Board (1024462) and all participants provided informed consent.

## Results

Across both phases of recruitment, a total of 41 caregivers and 36 youth consented to participate. Of the 77 users enrolled, 54% (22/41) of caregivers and 44% (16/36) of youth completed the post-study survey. Over 1500 text messages were sent in total during the study.

### Uptake and Willingness

Enrollment data during phase 1 showed that 63% (33/52) of youth approached about the study in the clinics agreed to participate. Reported reasons for declining included not being interested (7/19, 37%), not having a data plan or a cell phone (5/19, 26%), and caregivers not providing consent for participation (1/19, 5%). Reasons were not provided by 32% (6/19) of youth who declined. Among caregivers approached, 78% (29/37) agreed to participate in the study. Only 2 caregivers gave reasons for declining: one was not interested, and one did not have a data plan. Willingness to use the text service was 97% (75/77), with only 2 participants texting “STOP” before the end of the 6-week intervention period.

### Engagement and Satisfaction

**Figure 2:**
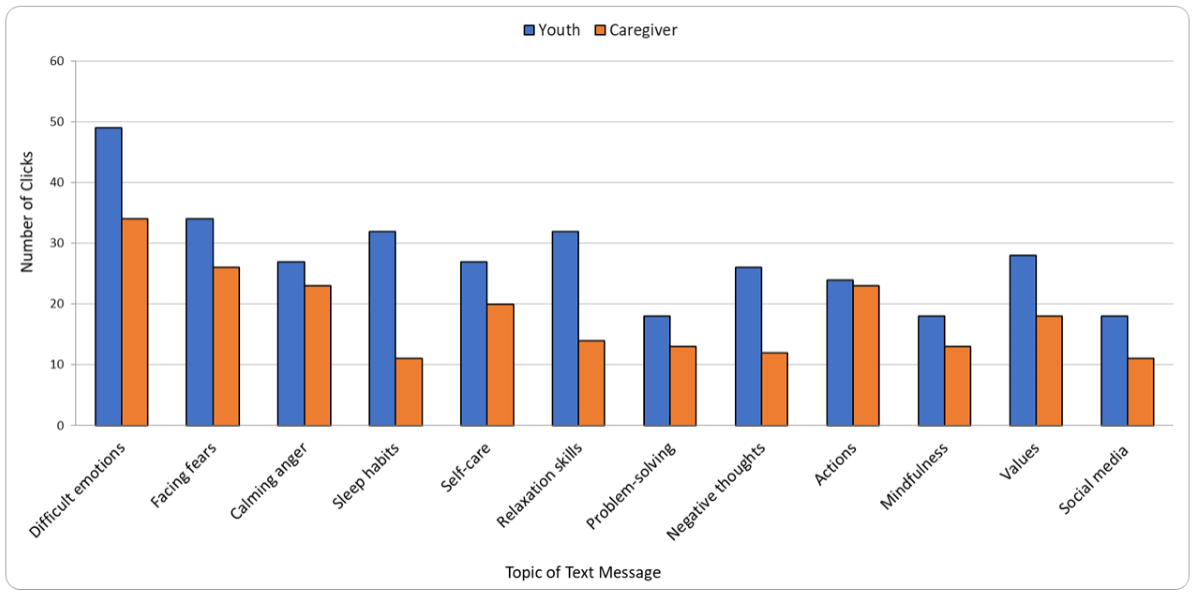
Total number of text message clicks by youth and caregivers across topics.

Overall, youth and caregivers opened links to resources and supports 551 times. Figure 2 shows the text message links clicked the most by youth, which were related to coping with difficult emotions (49 clicks; text message #1) and facing fears (34 clicks; text message #2). The linked resources clicked the most overall by caregivers were related to coping with difficult emotions (34 clicks; text message #1) and facing fears (26 clicks; text message #2). Overall, satisfaction with the text messages was positive, with 75% (12/16) of youth and 73% (16/22) of caregivers reporting they were “very satisfied“ or “slightly satisfied” (Table 2). Message frequency was highly satisfactory for both groups, with 81% (13/16) of youth and 86% (19/22) of caregivers saying that 2 messages a week worked well. Of the 16 youth, 2 (13%) thought it would have been better to receive more than 2 text messages a week.

**Table 2:** Comparison of youth and caregiver experiences with text messages.

Survey item			Number of responses by youth (N=16), n (%)		Number of responses by caregivers (N=22), n (%)
**Satisfaction**					
	Very satisfied	6 (38)		11 (50)	
	Slightly satisfied	6 (38)		5 (23)	
	Neutral	2 (13)		5 (23)	
	Slightly dissatisfied	2 (13)		1 (4)	
	Very dissatisfied	0 (0)		0 (0)	
**Frequency**					
	Timing worked for me	13 (81)		19 (86)	
	Should have been less often	1 (6)		3 (14)	
	Should have been more often	2 (13)		0 (0)	
**Helpfulness**					
	Very helpful	0 (0)		0 (0)	
	Helpful	7 (44)		13 (59)	
	Neutral	7 (44)		5 (23)	
	Somewhat helpful	2 (12)		4 (18)	
	Not at all helpful	0 (0)		0 (0)	
**Noticed personal change**					
	A lot of change	0 (0)		0 (0)	
	A fair amount of change	3 (19)		4 (18)	
	A bit of change	12 (75)		10 (45)	
	No change	1 (6)		8 (36)	
**Would recommend**					
	Yes	14 (88)		16 (73)	
	Maybe	1 (6)		6 (27)	
	No	1 (6)		0 (0)	
**Learned new information**					
	Yes	14 (88)		14 (63)	
	Maybe	1 (6)		4 (18)	
	No	1 (6)		4 (18)	
**Acted differently**					
	Yes	2 (12)		10 (45)	
	Maybe	8 (50)		6 (27)	
	No	6 (38)		6 (27)	
**Readied for next step**					
	Yes	7 (44)		5 (23)	
	Maybe	7 (44)		7 (32)	
	No	2 (12)		10 (45)	

### Value and Impact

Among youth completing the follow-up survey, 50% (8/16) indicated that the most helpful text message was the one related to coping with difficult emotions (text message #1). Three text messages were identified as the least helpful: healthy use of social media (4/16, 25%; text message #12), facing fears (4/16, 25%; text message #2), and personal values (4/16, 25%; text message #11). Fifty-six percent (9/16) of youth said the messages were “helpful” or “slightly helpful,” with an additional 44% (7/16) reporting a neutral response on helpfulness (Table 2). At the same time, 94% (15/16) of youth noticed at least some degree of change since receiving the text messages; 88% (14/16) indicated they had learned something new from the text messages, and 88% (14/16) would recommend it to someone they knew. Only one youth (1/16) provided an example when prompted to describe what they found themselves doing differently. The youth wrote “I was being more aware of myself and others. The timing of the text messages and my current life lined up exactly.”

Among caregivers completing the follow-up survey, there was less consensus as to the most helpful message. Nine of the 12 caregiver text messages were rated by at least one caregiver as the most helpful. The message focused on coping with difficult emotions (text message #1) was rated as the most helpful by 18% (4/22) of caregivers. The least helpful text messages, according to caregivers, were the ones about healthy social media use (6/22; 27%; text message #12) and sleep hygiene (4/22; 18%, text message #4). Among caregivers, 45% (10/22) reported acting differently since receiving the text messages, 63% (14/22) noticed at least some degree of change in themselves, and 63% (14/22) indicated they had learned something new from the text messages. When prompted to provide examples of the observed changes since receiving the text messages, caregivers pointed to a range of impacts. For example, “having more engaging conversations” with their teen, “keeping even tones when having difficult discussions,” “processing negative thoughts differently,” “managing anger,” and learning to “take a break from the conflict.” Seventy-three percent of caregivers (16/22) indicated they would recommend the text messages to people they knew. No youth or caregiver reported that the text messages were “very helpful,” but conversely, no youth or caregiver reported that the messages were “not helpful at all.”

When asked if the text messages had helped them feel ready for the next step in their treatment journey, 44% (7/16) of youth answered “yes,” and an additional 44% (7/16) answered “maybe.” Twenty-three percent of caregivers (5/22) said the text messages had helped them feel ready for their youth’s next step in the treatment journey, and 32% (7/22) answered “maybe.”

## Discussion

### Principal Findings

The intention of this pilot study was to develop and assess the feasibility of delivering a novel text-message campaign to engage youth clients and their caregivers during early stages of the mental health treatment pathway. As pilot testing is an almost essential prerequisite for a full-scale controlled study, this study helped test methods, learn more about the sample population, investigate recruitment strategies, and identify potential effects and associations that should be explored in a larger study [35].

Unlike other text-message interventions that have targeted a single clinical subpopulation (eg, those experiencing early psychosis) or desired behavior (eg, medication adherence), this study focused on transdiagnostic mental wellness and patient activation strategies for both youth and their caregivers. Our results suggest that for youth and caregivers new to outpatient mental health care and who are interested in gaining insights into mental wellness skills and strategies, a brief, time-limited text message intervention may be viable. These results bolster ongoing efforts to distill and integrate core mental wellness strategies that may be helpful across a variety of clinical conditions [36]. Theorizing about why certain text messages or linked resources were engaged with more than others was outside the scope of this pilot study but will be an important direction for future research to optimize and tailor the intervention to the needs and interests of the population. In the future, other avenues (eg, social media) for promoting and introducing transdiagnostic strategies, skills, and techniques could be explored.

The major finding of this study was that most youth and caregivers reported experiencing positive knowledge and personal change because of the intervention and found the text messages satisfactory. Enrollment rates and willingness to use the intervention were consistent with other text-messaging studies [37,38]. More than two-thirds of the youth (14/16, 88%) answered “yes” to questions asking if they learned something new from receiving the text messages, noticed a change due to receiving the text messages, and would recommend the text messages to others. Almost half (10/22, 45%) of the caregivers reported acting differently due to receiving the text messages. More than two-thirds (14/22, 63%) said they learned something new and would recommend the text messages to others. For a low-intensity intervention, these are not inconsequential real-life impacts. However, considerable work remains in the field of text-message interventions in general to refine and validate outcome measures that are sufficiently sensitive to changes (long-term and short-term) resulting from microinterventions like the brief series of text messages used here. Considering the minimal human resources required to deliver the automated services and the range of real-world changes participants reported (eg, managing anger better and having more engaging conversations between youth and caregivers), there is a significant opportunity to refine the intervention, refine the outcomes of interest, and leverage this modality in routine outpatient pathways in the future. Future research could examine whether early text-message support helps socialize youth and caregivers moving on to formal psychotherapy to the fact that therapeutic treatment requires active practice and application of skills, as opposed to something they passively receive. Text messages could even be trialed with individuals looking at e-mental health options online who choose not to enter the mental health system at that time.

Our findings may serve as a caution to future developers to avoid oversaturating youth and caregivers with too-frequent text messages. Two text messages a week over 6 weeks, a relatively brief campaign, was described as “timing worked for me” by 81% (13/16) of youth and 86% (19/22) of caregivers. The considerable variation in text messages identified as most and least useful suggests that technological capabilities would be useful to allow greater tailoring based on end-user preferences and concerns. While this study leveraged preexisting free online content, there could be advantages to cocreating locally relevant content that aligns with and reflects the preferences of the local community. Additionally, as we observed some downward trends in engagement over time, future studies could compare the order of the messages and the timing of delivery as features that could be optimized to promote ongoing engagement.

An unexpected finding, to be interpreted with caution given the small sample size, was that 45% of caregivers felt the text messages were not helpful for getting them ready for the next step of treatment. This may be because caregivers do not always perceive themselves to be part of the treatment and instead focus on their youth as the primary client. Caregivers may not have viewed their role in a mental health treatment journey as vital or something they needed to prepare for. It is also important to note that we did not measure which youth or caregivers would be coming back for further treatment (partnership appointments) and which had collaboratively decided that no further treatment was needed after the choice appointment. Future work could explore caregivers’ expectations of treatment pathways and how text messages could support transitions or further socialize caregivers to their vital role in the treatment success of youth. Collecting and analyzing data in caregiver-youth dyads could also help uncover important effects, interactions, and associations unique to particular family dynamics.

The study recruitment occurred in 2 time-limited phases during complex service and social changes due to COVID-19. It is unclear how that complexity may have impacted uptake, sense of helpfulness, views on which text messages were most valuable, or the ability of youth and caregivers to act on suggestions offered in the text messages (ie, suggested self-care strategies may have been impacted by physical distancing requirements or closures). In addition, phase 1 recruitment required clinicians to remember to introduce and enroll consenting youth and caregivers during a session. Future research may help illuminate the barriers to and enablers of clinician versus self-referral pathways and potential impacts on clinical workflow (eg, time and administration support needs). It is also important for future research to explore the possible mediating effects of clients’ personal clinicians introducing or promoting a text message intervention (phase 1) as opposed to individuals receiving a generic offer to enroll (phase 2).

### Limitations

Several limitations should be mentioned. First, although our follow-up survey response rate was consistent with the mean response rate of other youth mental health studies [39,40] a larger study, with statistical power to detect changes in both helpfulness and engagement, is required to confirm the effectiveness of the intervention. The small sample size, nonexperimental design, and short follow-up period prevent conclusions about any sustained behavior changes or the detection of significant differences between youth and caregivers’ usage and experiences. Second, to reduce the data entry burden on youth and caregivers, no baseline demographic data (eg, gender, symptom severity, or mental health literacy level) were collected, which limits our understanding of possible covariates. Third, youth and caregivers were sampled from an urban, community outpatient service and therefore may not represent broader youth and caregiver experiences. Finally, most outcome data were self-reported and subject to possible bias.

### Conclusions

Transdiagnostic mental wellness text messages for youth clients and caregiver engagement in community outpatient treatment are feasible. Although the sample size was small, the participants who followed through with the intervention had favorable impressions and reported promising changes in knowledge and behavior. Brief, skill-focused text messages may support youth and caregivers in experimenting with new ideas that could be helpful in making changes related to their mental health as a possible first step in community outpatient mental health services.

## Abbreviations

CAPA: choice and partnership approach
SMS: short messaging service
